# The prognostic value of right ventricular longitudinal strain in heart failure: a systematic review and meta-analysis

**DOI:** 10.1007/s10741-023-10329-y

**Published:** 2023-06-13

**Authors:** Vasileios Anastasiou, Andreas S. Papazoglou, Dimitrios V. Moysidis, Stylianos Daios, Dimitrios Tsalikakis, George Giannakoulas, Theodoros Karamitsos, Victoria Delgado, Antonios Ziakas, Vasileios Kamperidis

**Affiliations:** 1 First Department of Cardiology, Medical School, Aristotle University of Thessaloniki, AHEPA Hospital, St. Kiriakidi 1, Thessaloniki, 54636 GR Greece; 2 Department of Informatics and Telecommunication Engineering, University of Western Macedonia, Kozani, Greece; 3 Department of Cardiology, Hospital University Germans Triasi Pujol, Barcelona, Spain

**Keywords:** Speckle-tracking echocardiography, Heart failure, Right ventricular strain, Meta-analysis

## Abstract

**Background:** Right ventricular (RV) dysfunction is a well-recognized adverse prognostic feature in patients with heart failure (HF). Recently, many single-center studies have demonstrated that RV longitudinal strain assessed using speckle tracking echocardiography might be a powerful prognosticator in HF. **Objectives:** To systematically appraise and quantitatively synthesize the evidence of the prognostic value of echocardiographic RV longitudinal strain, across the entire spectrum of left ventricular ejection function (LVEF) in HF. **Methods:** A systematic literature review was conducted in electronic databases to identify every study reporting the predictive role of RV global longitudinal strain (RV GLS) and RV free wall longitudinal strain (RV FWLS) in HF subjects. A random-effects meta-analysis was conducted to quantify the adjusted and unadjusted hazard ratios [(a)HRs] for all-cause-mortality and for the composite outcome of all-cause mortality or HF-related hospitalization for both indices. **Results:** Twenty-four studies were deemed eligible and 15 of these provided appropriate quantitative data for the meta-analysis, encompassing 8,738 patients. Each 1% worsening in RV GLS and RV FWLS was independently associated with increased risk of all-cause mortality (pooled aHR = 1.08 [1.03–1.13]; p < 0.01; I^2^ = 76% and 1.05 [1.05–1.06]; p < 0.01; I^2^ = 0%, respectively) and the composite outcome (pooled aHR = 1.10 [1.06–1.15]; p < 0.01; I^2^ = 0% and 1.06 [1.02–1.10]; p < 0.01; I^2^ = 69%, respectively) for patients with HF. The subgroup analysis of HF patients with LVEF < 45% yielded similar results, with worsening in RV GLS and RV FWLS retaining strong association with the two outcomes. **Conclusion:** Echocardiographic RV GLS and RV FWLS appear to have powerful prognostic value across the range of HF.

**Supplementary Information:**

The online version contains supplementary material available at 10.1007/s10741-023-10329-y.

## Introduction

Right ventricular (RV) dysfunction is a well-known adverse prognostic feature in patients with heart failure (HF), as it commonly represents the phenotypic expression of advanced functional insult of the left ventricle, progressing to pulmonary hypertension and compromising of the RV mechanics [1]. Although a growing number of echocardiographic RV function indices have been proposed for diagnosing and risk stratifying patients with HF [2], accurate RV quantitative assessment is hindered by challenges to describe its complex anatomy and multilevel systolic contraction in a single measurement.

Two-dimensional speckle-tracking echocardiography has emerged as a novel imaging technique assessing the intrinsic myocardial function, and its applicability has recently been extended to the RV. Being less angle and geometry-dependent compared to conventional indices of RV function, RV longitudinal strain shows better inter- and intraobserver reproducibility, as compared to RV fractional area change, and allows for a more global assessment of intramyocardial contractile force. Both RV free wall longitudinal strain (RV FWLS) and RV global longitudinal strain (RV GLS) have been thoroughly investigated in different clinical settings [3, 4]. Although, their prognostic role has extensively been explored in diverse HF cohorts [5, 6], most studies are single-center with relatively small sample sizes [7, 8].

This review and meta-analysis sought to systematically appraise and quantitatively synthesize the existing evidence on the prognostic implications of RV GLS and RV FWLS across the entire spectrum of HF, reporting its association with the most clinically important outcomes; all-cause mortality and HF hospitalization.

## Methods

### Search strategy

The study was prospectively registered with the PROSPERO database (PROSPERO 2023 CRD42023383957). A systematic electronic search of published research up to January 18, 2023 was conducted using the MEDLINE, Scopus and Cochrane databases according to the Preferred Reporting Items for Systematic Reviews and Meta-Analyses guidelines [9]. The Medical Subject Headings and keywords used as search terms were: (‘’right ventricular free wall longitudinal strain’’ OR ‘’right ventricular global longitudinal strain’’ OR ‘’right ventricular strain’’) and (‘’heart failure’’) and (‘’outcome’’ OR ‘’prognosis’’). The reference lists of the included studies and relevant reviews were also hand-searched to identify further relevant studies.

### Study selection – eligibility criteria

The eligibility of the retrieved studies was independently assessed by two investigators (V.A., S.D.), according to prespecified criteria. Studies investigating the prognostic significance of RV GLS or RV FWLS (measured either as dichotomous or as continuous variable) in patients with HF, irrespective of left ventricular ejection fraction (LVEF) were included in this systematic review. Abstracts without complete published papers, case reports, review papers, editorials, and letters were excluded. Studies not reporting hazard ratios (HRs) and studies in which RV GLS and RV FWLS were assessed solely as dichotomous variables in the Cox Regression analysis were not included in the meta-analysis. Any discrepancies were resolved by consensus or by the involvement of a third reviewer (D.V.M). Overall synthesis and reporting of this systematic review and meta-analysis are in line with the Preferred Reporting Items for Systematic Reviews and Meta-Analyses guidelines (Table S1 of the supplementary appendix) [9].

### Risk of bias of individual studies

The methodological quality of the individual studies was assessed independently by two investigators (V.A. and S.D.) using the Quality in Prognosis Studies tool [10]. The risk of bias for each eligible study was evaluated in each of the following domains as “low”, “moderate” or “high”: study participation, study attrition, prognostic factor measurement, outcome measurement, study confounding, statistical analysis and reporting. The possibility of publication bias was not evaluated through the funnel plot method described by Egger and colleagues due to the limited number of eligible studies [11].

### Outcomes of interest

The primary study outcome was all-cause mortality, while the secondary composite study outcome was the occurrence of either all-cause death or any HF-related hospitalization (including acute HF admission, heart transplantation, left ventricular assist device implantation).

### Data synthesis and statistical analysis

All adjusted and unadjusted HRs and the corresponding 95% confidence intervals [12] were extracted for the continuous RV GLS and RV FWLS variables to reflect the risk difference per 1% (1%) worsening in RV GLS and RV FWLS. Pooled adjusted HRs and 95% CIs were computed using random-effect models (DerSimonian and Laird method) on the association of RV GLS and RV FWLS with the defined outcomes of interest, adjusted or unadjusted for clinical differences between the populations. A random effects model was selected a priori given the expected heterogeneity in study design across the eligible studies.

Separate analysis using only unadjusted or adjusted data was conducted. Subgroup analysis was performed using only studies including patients with LVEF < 45%. Forest plots were constructed to show the overall effect of each parameter. The observed heterogeneity in each analysis was described using the I^2^ statistic, which was quantified as low (< 25%), moderate (25–75%), or high (> 75%) [13]. All statistical analyses were performed using Review Manager (RevMan), Version 5.4. (2020), with 2-tailed p-values of less than 0.05 indicating statistical significance.

## Results

### Study selection and baseline characteristics

**Fig. 1:**
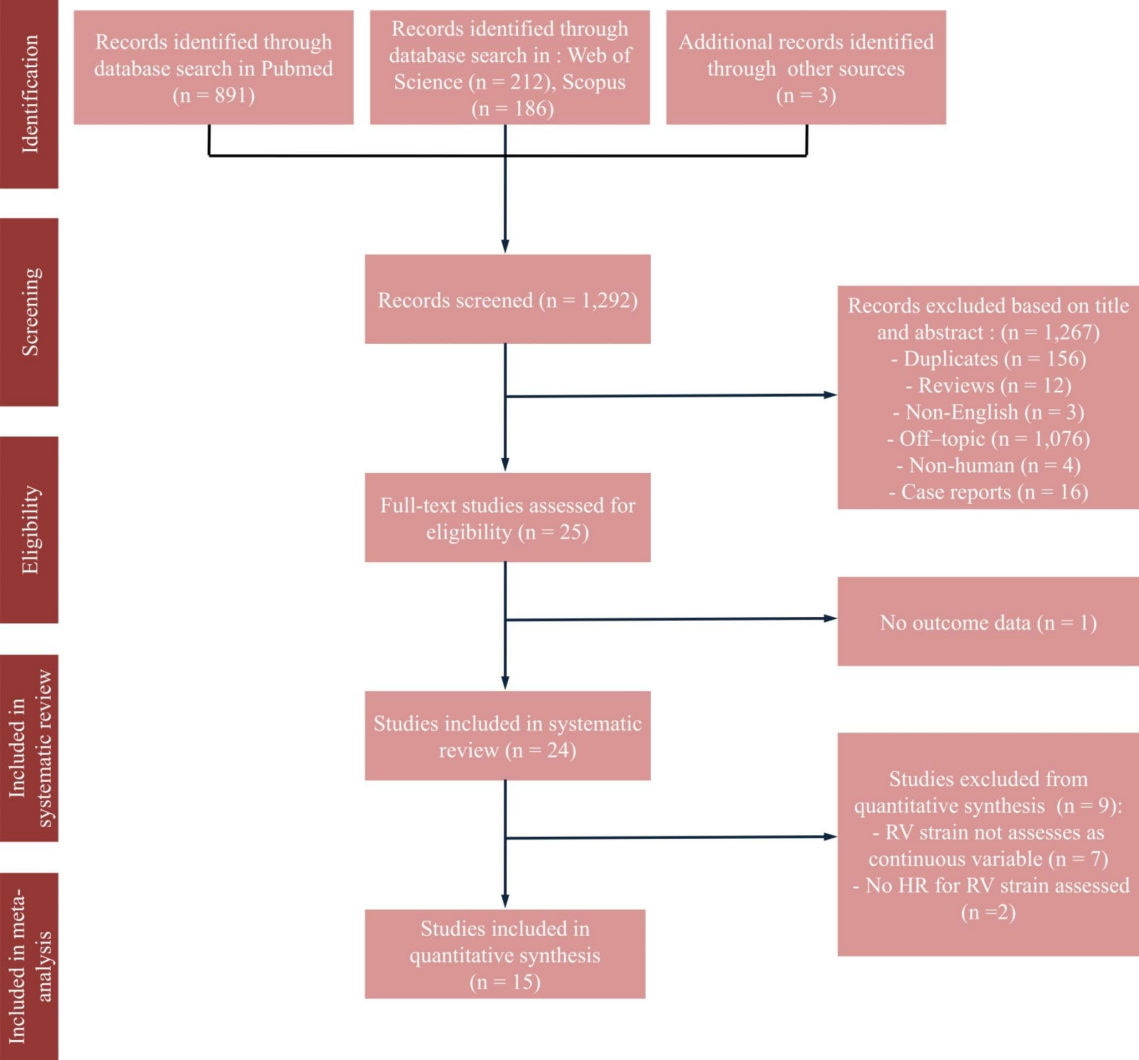
Flowchart of the Study Selection Process HR, hazard ratio; RV right ventricle

The process of study selection is summarized in Fig. 1. From the initial 1,292 studies identified based on the search strategy, 24 relevant eligible full-text articles were included [5–8, 14–33]. Nine of the studies were excluded from the meta-analysis due to unreported HR, or because outcomes for RV strain were not assessed as continuous variables [14, 16–20, 23, 26, 33]. Overall, the risk of bias was considered to be low or moderate in the included studies (Table S2 of the supplementary appendix). Six studies were considered of moderate quality, mainly driven by moderate risk of bias in study participation, study attrition, prognostic factor measurement and study cofounding domains [7, 8, 28, 30–32].

The baseline characteristics of the 24 included studies are summarized in Table 1. All studies were observational, with the majority of studies being prospective cohorts and only 6 studies being retrospective. The overall follow-up ranged from 7.5 (range; 1.5–13.5) to 97 (range; 53–145) months. In terms of population characteristics 11 studies included only patients with HF with reduced EF (HFrEF) [5, 7, 8, 14, 17, 18, 25, 28–30, 32], 2 studies included HFrEF and HF with mildly reduced EF patients (HFmrEF) [20, 21], in 1 study patients had only HF with preserved EF (HFpEF) [26], and the rest 10 included the whole range of LVEF [6, 15, 16, 19, 22–24, 27, 31, 33]. Four studies investigated solely subjects with acute decompensated HF [6, 14, 23, 33], while the rest included clinically stable HF patients or mixed cohorts. Ten of the included studies reported data on both RV GLS and RV FWLS [7, 8, 16, 19, 21, 25, 26, 29–31], 11 studies assessed only RV FWLS [5, 14, 17, 20, 22–24, 27, 28, 32, 33], and 3 studies evaluated only RV GLS [6, 15, 18]. Most studies used General Electric (GE) Healthcare (n = 16) for the echocardiographic analysis, while 2 used only Phillips and 3 used other vendors.

**Table 1:** Baseline characteristics of included studies

Author	Year	Design	No. of patients	Age, years	Male, %	HF population	LVEF cut-off for inclusion, %	LVEF, %	RV strain index	Vendor	Follow-up period, months
Verhaert et al. [14]	2010	Prospective	62	56 ± 13	79	Acute	≤ 35	26 ± 10	RV FWLS	Not specified	7.5 (range; 1.5–13.5)
Guendouz et al. [15]	2012	Prospective	104	57 ± 11	83	Stable	All	28 ± 8	RV GLS	GE	37 (range; 23–51)
Cameli et al. [16]	2013	Prospective	98	59 ± 8	62	Stable, advanced HF	All	26.4 ± 4.1	RV FWLS and RV GLS	GE	18 (range; 7.2–28.8)
Vizzardi et al. [17]	2014	Prospective	60	60.2 ± 10.1	83	Stable	< 40	29.8 ± 8.5	RV FWLS	GE	40 (range; 26.8–53.2)
Park et al. [18]	2014	Retrospective	72	64 ± 12	75	Ischaemic heart disease	< 40	27.6 ± 9	RV GLS	GE, Phillips	15 (range; 6–24)
Motoki et al. [8]	2014	Prospective	171	57 ± 14	73	Stable	≤ 35	25 ± 6	RV FWLS and RV GLS	Not specified	60
Garcia-Martin et al. [19]	2015	Prospective	103	72.9 ± 14.4	35	Stable	All	64.3 ± 13.9	RV FWLS and RV GLS	GE	23.1 (range; 10.7–35.5)
Sciatti et al. [20]	2015	Retrospective	60	60 ± 10	83.3	Stable	≤ 45	30 ± 9	RV FWLS	GE	32 (range; 19–45)
Iacovielloet al. [21]	2016	Prospective	332	64 ± 14	76	Stable	< 45	33 ± 9	RV FWLS and RV GLS	GE	36 (range; 10–62)
Bosch et al. [22]	2017	Prospective	657	68 ± 11 for HFpEF 65 ± 11 for HFrEF	50.7	Acute and stable	All	59 ± 6 - HFpEF31 ± 10 - HFrEF	RV FWLS	GE	47.7 (median)
Park et al. [6]	2018	Retrospective	1824	70.4 ± 13.8	53	Acute	All	39.3 ± 15.2	RV GLS	GE, Siemens, Philips	31.7 (range; 11.6–54.4)
Hamada-Harimura et al. [23]	2018	Prospective	618	72 ± 13	62	Acute	All	46 ± 16	RV FWLS	GE, Phillips, Toshiba	14.2 (range; 6.8–23.5)
Carluccio et al. [5]	2018	Prospective	200	66 ± 11	76	Stable	< 40	30 ± 5	RV FWLS	GE	28 (range; 13–44)
Carluccio et al. [7]	2019	Prospective	288	66 ± 11	77	Stable	< 40	30 ± 5	RV FWLS and RV GLS	GE	23.8 (range; 11.6–41.8)
Prihadi et al. [24]	2019	Retrospective	896	71 ± 8	51.3	Significant functional TR	All	46.1 ± 14.9	RV FWLS	GE	33.6 (range; 15.6–64,8)
Houard et al. [25]	2019	Prospective	266	60 ± 14	79	Stable	< 35	23 ± 7	RV FWLS and RV GLS	Philips	56.4 (median)
Lejuene et al. [26]	2020	Prospective	149	78 ± 9	39	HFpEF	> 50	63 ± 7	RV FWLS and RV GLS	Phillips	30 (range; 21–39)
Gavazzoni et al. [27]	2020	Prospective	458	60 ± 13	65	Stable	All	44 ± 14	RV FWLS	GE	64.8 (range; 50.4–79.2)
Ishiwata et al. [28]	2021	Retrospective	109	44 ± 14	69.7	DCM	< 40	21.9 ± 7.3	RV FWLS	Not specified	12
Vijiiac et al. [29]	2021	Prospective	50	61 ± 14	68	DCM	< 40	25 ± 7	RV FWLS and RV GLS	GE	16 (range; 13–19)
Lundorff et al. [30]	2021	Retrospective	701	66.1 ± 10.8	62	Stable	< 40	25.3 ± 8.2	RV FWLS and RV GLS	GE	39 (range; 21–56)
Ancona et al. [31]	2021	Prospective	171	74.3 ± 10.2	36.8	Severe TR	All	52.2 ± 12.7	RV FWLS and RV GLS	GE	30
Stassen et al. [32]	2022	Prospective	871	64.9 ± 10.7	74.5	CRT recipients	< 35	27.5 ± 8.1	RV FWLS	GE	97 (range; 53–145)
Berril et al. [33]	2022	Prospective	418	-	-	Acute	All	-	RV FWLS	GE	24

Age and LVEF are reported as mean ± standard deviation

CRT, cardiac resynchronization therapy; DCM, dilated cardiomyopathy; GE, general electric healthcare; HF, heart failure; HFpEF; heart failure preserved ejection fraction; HFrEF, heart failure reduced ejection fraction; LVEF, left ventricular ejection fraction; RV, right ventricle; RV FWLS, right ventricular free wall longitudinal strain; RV GLS, right ventricular global longitudinal strain; TR, tricuspid regurgitation

### Predictive value of RV strain: all-cause mortality

**Fig. 2:**
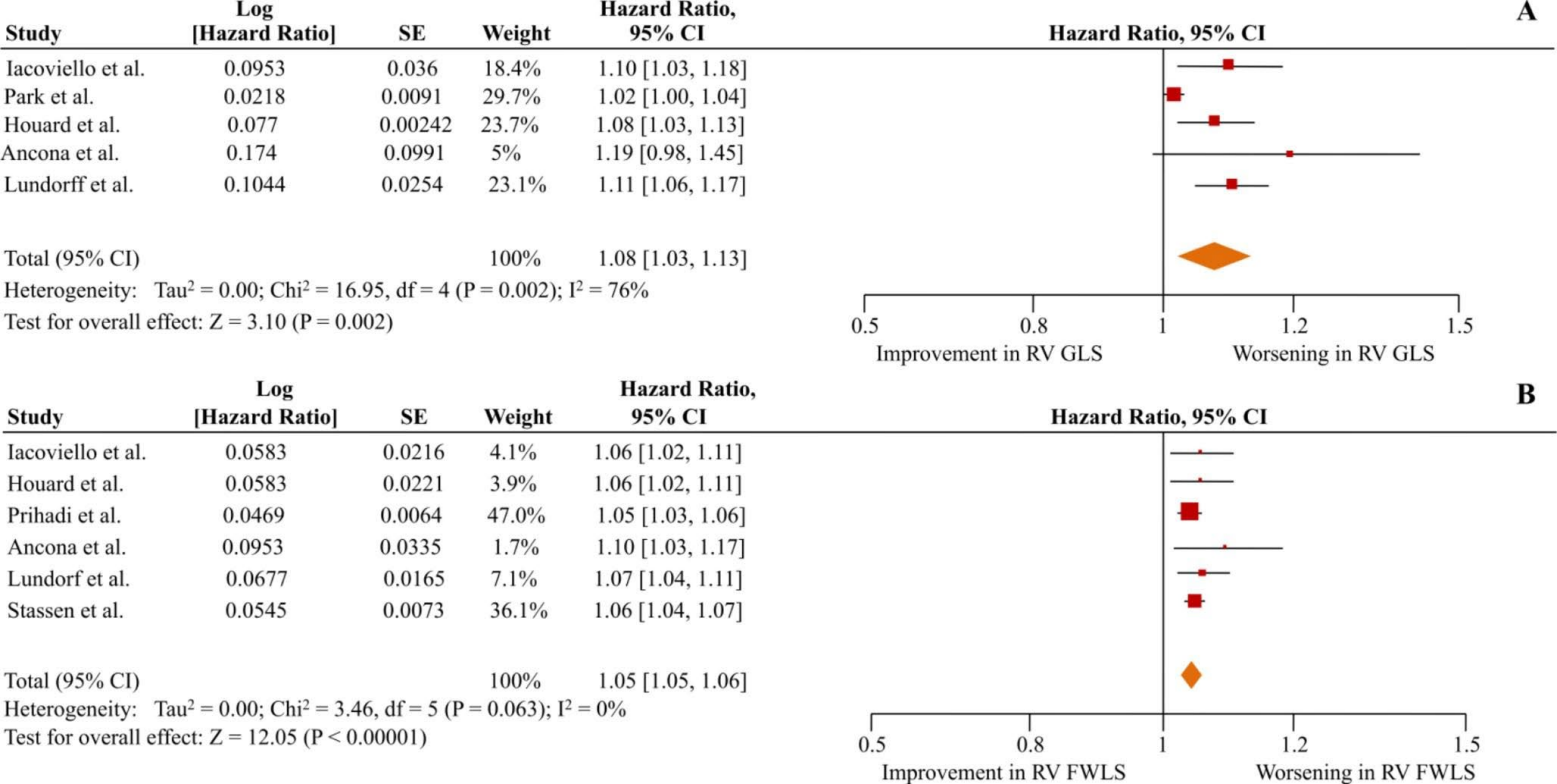
Right ventricular global longitudinal strain **(**RV GLS) and right ventricular free wall longitudinal strain (RV FWLS) as predictors of all-cause mortality in heart failure patients irrespective of left ventricular ejection fraction. The forest plots display the adjusted hazard ratios and 95% confidence intervals (CI) for the association of RV GLS (upper panel) and RV FWLS (lower panel) per 1% worsening with all-cause mortality for all heart failure patients

**Fig. 3:**
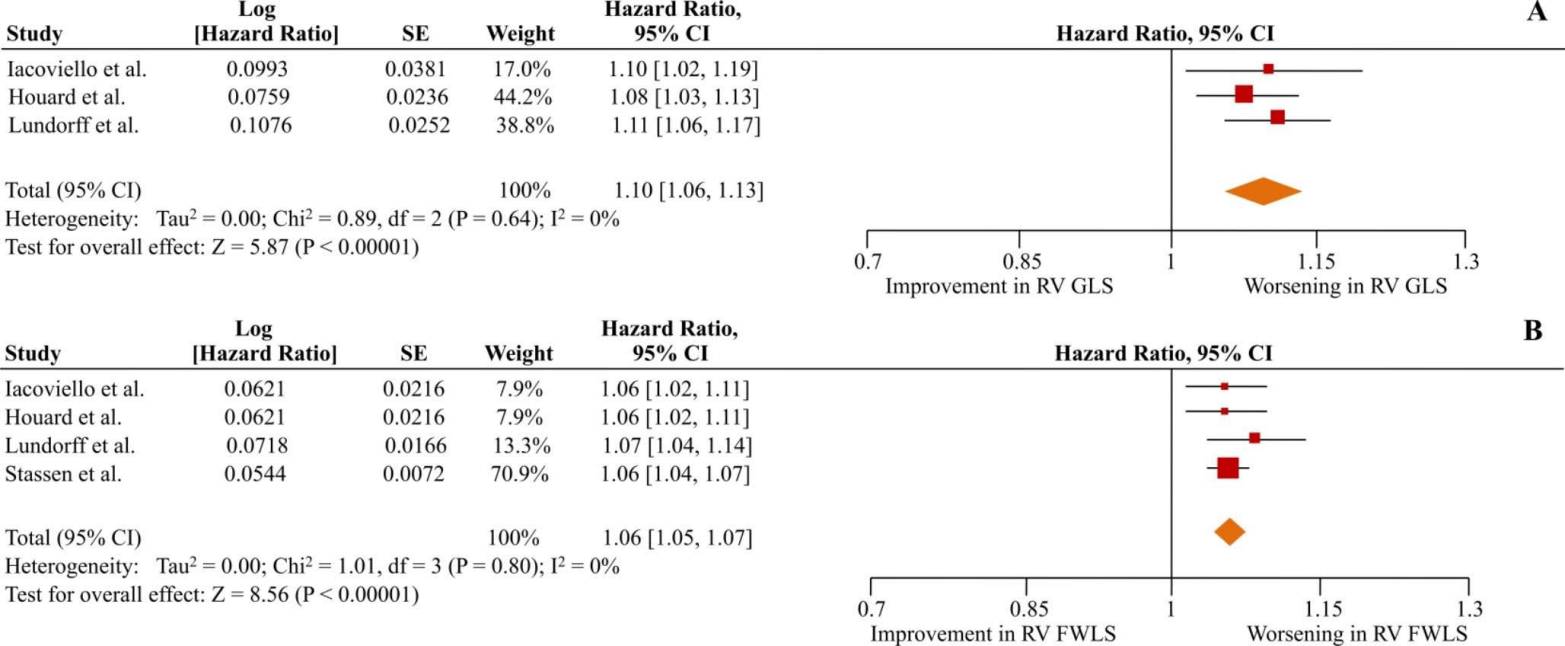
Right ventricular global longitudinal strain (RV GLS) and right ventricular free wall strain (RV FWLS) as predictors of all-cause mortality in heart failure patients with left ventricular ejection fraction < 45%. The forest plots display the adjusted hazard ratios per 1% worsening and 95% confidence intervals (CI) for increasing association of RV GLS (A) and RV FWLS (B) with all-cause mortality for patients with heart failure and left ventricular ejection fraction < 45%

Regarding the primary outcome of all-cause mortality, the unadjusted pooled HRs were 1.09 (1.04–1.16; p < 001;I^2^ = 91%) per 1% worsening of RV GLS and 1.07 (1.04–1.09; p < 0.01;I^2^ = 71%) per 1% worsening of RV FWLS, as depicted in Figure S1 of the supplementary appendix. When adjusted for pre-specified clinically-relevant parameters, it was shown that for each unit of worsening in RV GLS and RV FWLS the risk for all-cause death was increased by 8% and 5% (adjusted HR = 1.08 [1.03–1.13]; p < 0.01; I^2^ = 76% and 1.05 [1.05–1.06]; p < 0.01; I^2^ = 0%), respectively (Fig. 2). The results were similar when sub-analysis in patients with LVEF < 45% was performed, synthesizing data from 3 studies for RV GLS [21, 25, 30] (adjusted HR = 1.10 [1.06–1.13]; p < 0.01; I^2^ = 0), and from 4 studies for RV FWLS [21, 25, 30, 32] (adjusted HR = 1.06 [1.05–1.07]; p < 0.01; I^2^ = 0) (Fig. 3).

### Predictive value of RV strain: composite outcome of all-cause mortality or any HF-related hospitalization

**Fig. 4:**
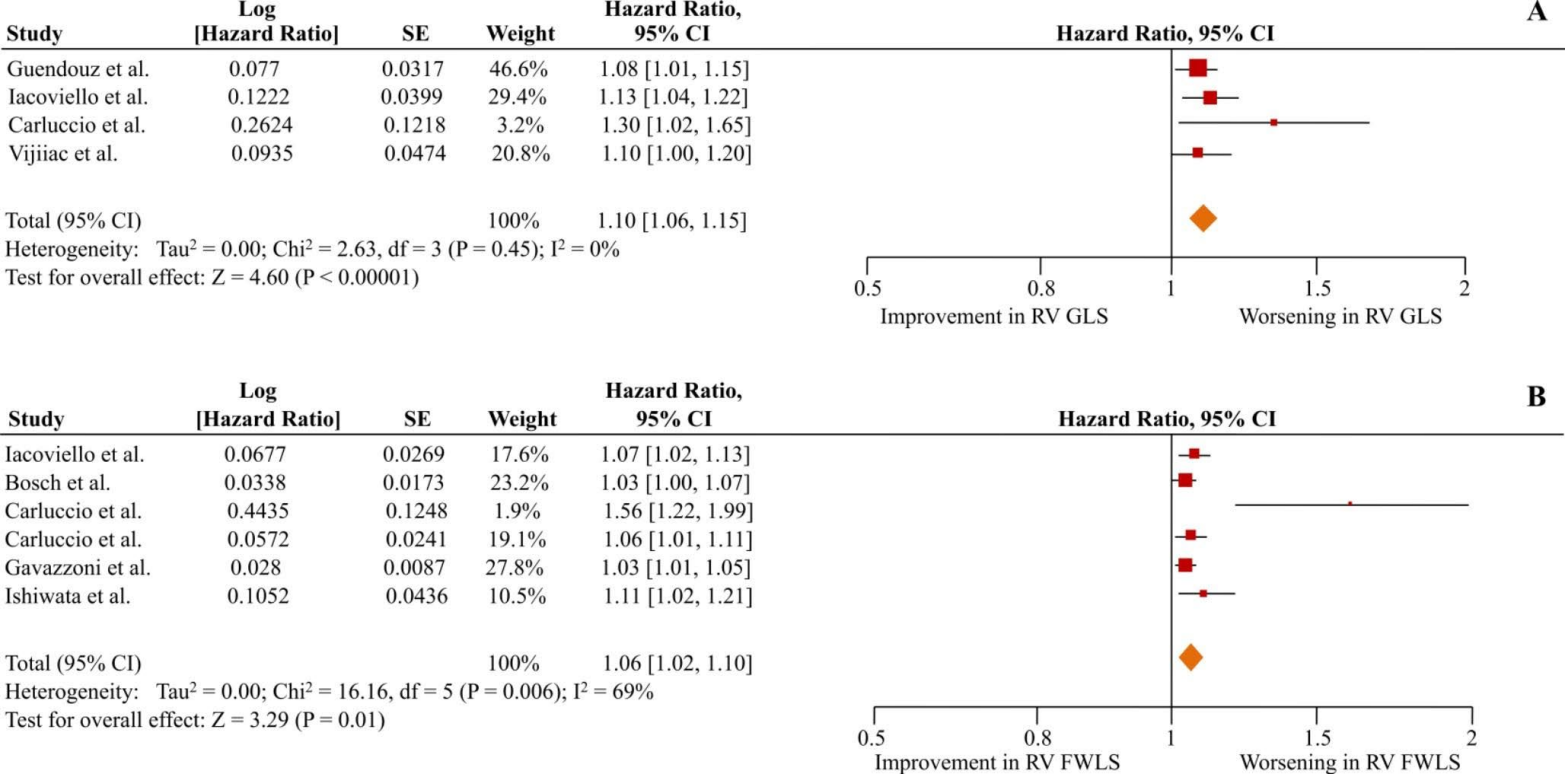
Right ventricular global longitudinal strain (RV GLS) and right ventricular free wall strain (RV FWLS) as predictors of the composite outcome of all-cause mortality or any heart failure (HF)-related hospitalization in HF patients irrespective of left ventricular ejection fraction. The forest plots display the adjusted hazard ratios per 1% worsening and 95% confidence intervals (CI) for increasing association of RV GLS (A) and RV FWLS (B) with the composite outcome of all-cause mortality or any HF-related hospitalization for HF patients irrespective of left ventricular ejection fraction

**Fig. 5:**
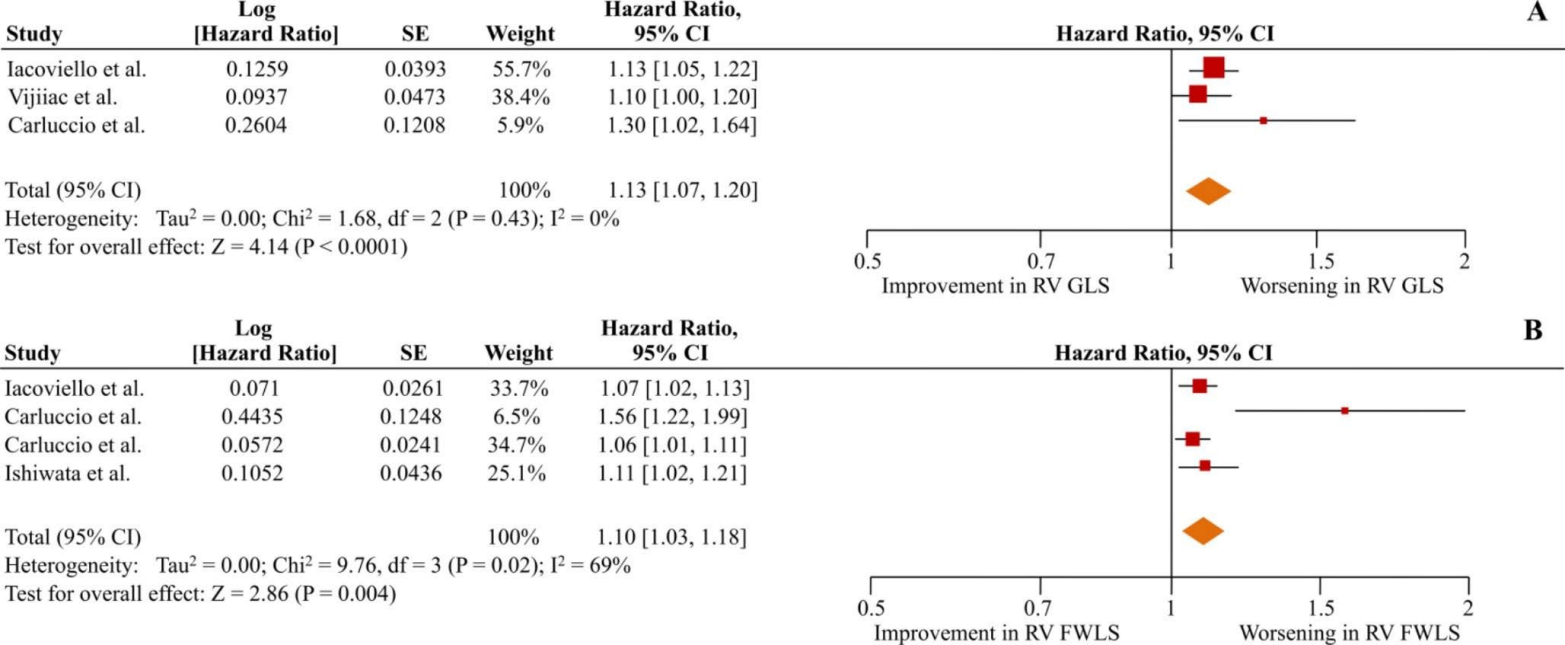
Right ventricular global longitudinal strain (RV GLS) and right ventricular free wall strain (RV FWLS) as predictors of the composite outcome of all-cause mortality or any heart failure (HF)-related hospitalization in HF patients with left ventricular ejection fraction < 45%. The forest plots display the adjusted hazard ratios per 1% worsening and 95% confidence intervals (CI) for increasing association of RV GLS (A) and RV FWLS (B) with the composite outcome of all-cause mortality or any HF-related hospitalization for HF patients with left ventricular ejection fraction < 45%

The unadjusted pooled HRs of the composite secondary outcome of all-cause death or any HF-related hospitalization were 1.22 (1.10–1.37; p < 0.01; I^2^ = 87%) and 1.10 (1.05–1.16; p < 0.01; I^2^ = 88%) per 1% worsening of RV GLS and RV FWLS respectively (Figure S2 in the supplementary appendix). Four studies reported adjusted data for the secondary outcome for RV GLS [5, 15, 21, 29], and 6 studies for RV FWLS [5, 7, 21, 22, 27, 28]. In patients with HF irrespective of LVEF, each 1% worsening in RV GLS and RV FWLS was associated with a 10% and 6% risk of the occurrence of the secondary outcome, respectively (adjusted HR = 1.10 [1.06–1.15]; p < 0.01; I^2^ = 0% and HR = 1.06 [1.02–1.10]; p < 0.01; I^2^ = 69%) (Fig. 4). The subgroup analysis of patients with LVEF < 45% yielded similar results (pooled adjusted HR = 1.13 [1.07–1.20]; p < 0.01; I^2^ = 0% for RV GLS and 1.10 [1.03–1.18]; p < 0.01, I^2^ = 69% for RV FWLS) (Fig. 5).

### Optimal cut-off values of RV longitudinal strain to predict outcomes

**Fig. 6:**
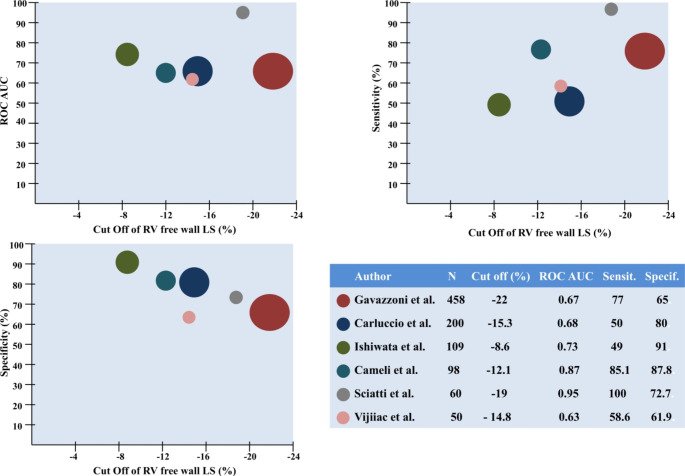
Predictive value of right ventricular free wall longitudinal strain (RV FWLS) for the composite outcome of all-cause mortality or any heart failure-related hospitalization in heart failure patients The size of the bubbles is indicative of the number of patients in each study. AUC, area under the curve; ROC, receiver-operating characteristic

Figure 6 depicts the ability of RV FWLS to predict the composite end-point of all-cause mortality or any HF-related hospitalization across different HF cohorts. Six studies reported the results of receiver-operating characteristic curve analysis for RV FWLS [5, 16, 20, 27–29]. Substantial heterogeneity was observed regarding suggested optimal cutoff values (-22 to -8.6%), and their respective predictive capacity [area under the curve (0.63 to 0.95), sensitivity (49–100%), and specificity (61.9–87.8%)] for the composite outcome depending on the HF-subtype under investigation.

## Discussion

The present study was a systematic review and meta-analysis of 24 studies comprising a total of 8,738 patients, which assessed the prognostic role of echocardiography-derived RV longitudinal strain in HF. Its main finding is that RV FWLS and RV GLS were strong predictors of adverse outcomes in HF. Both indices retained independent association with all-cause mortality and the composite end-point of all-cause mortality or any HF-related hospitalization even after adjustment for clinically relevant characteristics. When subgroup analysis was performed for patients with LVEF < 45% the association with events remained significant for both RV indices.

### The value of RV dysfunction in HF

Numerous pathophysiological pathways may impair RV function in left sided heart disease. In HF, RV dysfunction is commonly the sequelae of pulmonary venous hypertension secondary to *de novo* elevation in LV end-diastolic pressure, being backwards transmitted to the pulmonary vascular bed [34]. Chronic elevation of the LV filling pressure due to LV systolic or diastolic dysfunction in HF is passively backwards transmitted to the left atrium. Elevated left atrial pressure is upstream transmitted to the pulmonary vasculature causing pulmonary vascular remodeling leading to pulmonary hypertension. These elevated pulmonary pressures are transferred to the thin-walled flow-generator RV which is not designed to cope with brisk increases of afterload [35]. Although at the first compensatory phase the RV adapts to the pressure overload by myocyte hypertrophy and augmented contractility, it eventually undergoes adverse remodeling and chamber dilation [36]. The RV dilatation leads to tricuspid annulus dilatation and tethering of the tricuspid leaflets, begets functional tricuspid regurgitation, which causes RV volume overload, further exacerbating RV remodeling and RV dysfunction [37]. Passed this point, HF patients enter a vicious cycle of recurrent HF admissions and exhibit a particularly malignant prognostic course. Other causes of RV dysfunction, such as RV infarction in ischaemic HF, intrinsic RV myocardial disarray in non-ischaemic cardiomyopathy, and RV involvement in myocardial infiltrative diseases can also lead to clinical RV failure [38].

Irrespective of the primary cause, resultant RV impairment conveys advanced disease progression and portends dismal prognosis [39, 40]. Therefore, diagnosis of RV dysfunction should be established with ease in a reproducible manner in clinical practice. The quest for an RV function index that describes global RV performance, combining those features has stimulated the development of new imaging techniques, such as strain imaging.

### Strain imaging compared to conventional echocardiographic RV assessment

Echocardiography remains a valuable modality for RV assessment, offering widespread availability, portability, and ease of use. Various echocardiographic indices of prognostic relevance have been identified in HF to assess RV function. Tricuspid annular plane excursion measured by M-mode is an index that has dominated clinical practice for RV assessment for decades with recognized prognostic utility in HF [41, 42]. However, it has inherent limitations; including its angle-dependency, the single-plane nature of M-mode, and the extrapolation of a single segment to reflect overall RV performance. Those characteristics render M-mode less sensitive than speckle-tracking to detect RV dysfunction [7]. Carluccio et al. demonstrated that among 200 HFrEF patients with preserved RV function by tricuspid annular plane excursion, RV FWLS could identify a subgroup with impaired longitudinal RV function and an adjusted 2-fold increased risk of events [7]. This finding exemplifies that RV strain can detect RV dysfunction at an earlier stage compared to tricuspid annular plane systolic excursion in HF patients.

### Prognostic impact of RV strain in HF subgroups

Although most studies in this meta-analysis included stable, outpatients with HF [25, 27] a few studies have addressed the importance of RV strain in the acute HF setting [6, 23]. Park et al. studied the largest cohort of hospitalized patients with acute HF including a total of 1,824 subjects, and concluded that RV GLS was a powerful predictor of all-cause mortality irrespective of LV function, evaluated by LV GLS [6]. Moreover, patients with impaired LV GLS and RV GLS exhibited the worst outcome [6]. Hamada-Harimura et al. showed that RV FWLS was an independent associate of outcomes for both HFrEF and HFpEF patients with acute decompensated HF, while LV GLS failed to elicit prognostic information [23].

HFrEF represents a challenging population in terms of risk stratification and therapeutic decision making. Several studies have investigated the prognostic implications of RV strain imaging on subjects with reduced EF [5, 7, 8, 18, 20, 21, 25, 32]. Stassen et al. focused on HFrEF patients who had received cardiac resynchronization therapy and demonstrated that RV FWLS could identify high-risk individuals and provided incremental prognostic information over traditional echocardiographic parameters of RV dysfunction [32]. Cameli et al. studied patients with more advanced HF, referred for transplantation and indicated that RV FWLS was the strongest outcome predictor among indices of both LV and RV function [16]. As suggested by the results of this meta-analysis, RV strain may equally serve as an effective imaging biomarker for the subgroup of HF patients with impaired systolic function (Figs. 3 and 5).

Setting an optimal cut-off value of RV strain based on current literature is challenging due to considerable heterogeneity of the HF populations being examined in various studies. For patients with dilated cardiomyopathy and reduced LVEF or advanced HF optimal cut-offs of RV FWLS to predict events fall into a more impaired category [16, 28]. On the contrary, more preserved RV FWLS values are suggested when patients with mildly impaired LVEF [20] or preserved LVEF [27] are included. Nonetheless different RV FWLS cut-off values can accurately predict events in different HF populations with the range of area under the curve varying from 0.63 to 0.95, with sensitivity of 49–100% and specificity of 65 to 91% (Fig. 6). On the basis of these findings, RV strain imaging is a valuable risk stratification tool applicable for all HF phenotypes starting from early disease stages to end-stage HF.

### Limitations

Some limitations of this study should be acknowledged. First of all, as the nature of all studies included is observational, variations in the inclusion criteria and endpoints are all potential sources of heterogeneity among studies, despite the strict study selection protocol with robust methodology. In specific, heterogeneity expands to the type of HF cohorts included (i.e. chronic or acute HF, HF with reduced or preserved LVEF), the number of patients in each study, as well as the follow up period. Furthermore, although the variables used for multivariate adjustment across the included studies coincide to some extent, they are not identical; hence pooled HRs should be interpreted with caution.

Moreover, this meta-analysis could not prove the additive prognostic value of RV longitudinal strain parameters over conventional indices of RV function due to the lack of available patient-level data to enable direct meta-analytic comparisons. In like manner, a direct comparison of the predictive value between RV GLS and RV FWLS was unfeasible due to lack of available published diagnostic accuracy data of the two variables for the same outcome measurement.

Additionally, meta-regression analysis could not be performed because of the limited number of eligible studies. Finally, intervendor and intersoftware standardization is a major pitfall of speckle-tracking echocardiography and even though 16 studies solely utilized one vendor, a few studies provided mixed data from different vendors.

## Conclusion

RV strain indices, RV GLS and RV FWLS, are novel noninvasive imaging biomarkers that can be routinely assessed in all HF patients and appear to be robust outcome predictors for diverse subgroups of the HF spectrum. Further research is warranted to allow integrating those parameters in risk stratification models and translate their predictive value into clinical decision making in HF.

### Electronic supplementary material

Below is the link to the electronic supplementary material.

Supplementary Material 1

## Abbreviations

CIs: Confidence intervals
FWLS: Right ventricular free wall longitudinal strain
GLS: Right ventricular global longitudinal strain
HF: Heart failure
HFmrEF: Heart failure with mildly reduced ejection fraction
HFpEF: Heart failure with preserved ejection fraction
HFrEF: Heart failure with reduced ejection fraction
HR: Hazard ratio
LV: Left ventricular
LVEF: Left ventricular ejection fraction
RV: Right ventricle/ventricular
