# Supplementary material for: The prognostic value of right ventricular longitudinal strain in heart failure: a systematic review and meta-analysis
Source: Heart Fail Rev. 2023 Jun 13;28(6):1383–94. doi: 10.1007/s10741-023-10329-y (PMC10575809; doi:10.1007/s10741-023-10329-y)
Supplement: Supplementary file 1 — Supplementary Material 1 [file 10741_2023_10329_MOESM1_ESM.docx]

**Declarations**

Ethical Approval

Non-applicable, it is a meta-analysis and systematic review

Competing interests

Vasileios Anastasiou, Andreas S. Papazoglou, Dimitrios V. Moysidis, Styliannos Daios, Dimitrios Tsalikakis, George Giannakoulas, Theodoros Karamitsos, Antonios Ziakas, and Vasileios Kamperidis have nothing to disclosure.

Victoria Delgado received speaker fees from Edwards Lifesciences, Medtronic, Novartis and Philips and consulting fees from Edwards Lifesciences and Novo Nordisk.

Authors' contributions

Vasileios Anastasiou: generate the concept of the meta-analysis, searching the literature, statistical analysis, drafting the manuscript

Andreas S. Papazoglou: statistical analysis, drafting the manuscript

Dimitrios V. Moysidis: searching the literature, statistical analysis

Stylianos Daios: searching the literature, statistical analysis

Dimitrios Tsalikakis: statistical analysis

George Giannakoulas: revise the intellectual content and provide critical review

Theodoros Karamitsos: revise the intellectual content and provide critical review

Victoria Delgado: revise the intellectual content and provide critical review

Antonios Ziakas: drafting the manuscript, revise the intellectual content and provide critical review

Vasileios Kamperidis: generate the concept of the meta-analysis, statistical analysis, drafting the manuscript, revise the intellectual content and provide critical review

Funding

None

Availability of data and materials

All datasets will be accessible by the *Journal* upon request.
